# Serum Cystatin C, Markers of Chronic Kidney Disease, and Retinopathy in Persons with Diabetes

**DOI:** 10.1155/2015/404280

**Published:** 2015-10-20

**Authors:** Chee Wai Wong, Boon Wee Teo, Ecosse Lamoureux, Mohammad Kamran Ikram, Jie Jin Wang, E. Shyong Tai, Sunil Sethi, Tien Yin Wong, Charumathi Sabanayagam

**Affiliations:** ^1^Singapore Eye Research Institute, Singapore National Eye Centre, The Academia, 20 College Road, Discovery Tower Level 6, Singapore 169856; ^2^Department of Medicine, Singapore National University Hospital, National University of Singapore, 5 Lower Kent Ridge Road, Singapore 119074; ^3^Ophthalmology and Visual Sciences Academic Clinical Program, Duke-NUS Graduate Medical School, 8 College Road, Singapore 119074; ^4^Department of Ophthalmology, Singapore National University Hospital, National University of Singapore, 5 Lower Kent Ridge Road, Singapore 119074; ^5^Westmead Millennium Institute for Medical Research, C24 Westmead Hospital, University of Sydney, Sydney, NSW 2006, Australia; ^6^Department of Pathology, Singapore National University Hospital, National University of Singapore, 5 Lower Kent Ridge Road, Singapore 119074

## Abstract

*Purpose.* We examined the association of CKD defined by serum creatinine, serum cystatin C, and albuminuria with moderate diabetic retinopathy (DR). *Methods.* We examined 1,119 Indian adults with diabetes, aged 40–80 years, who participated in the Singapore Indian Eye Study (2007–2009), a population-based cross-sectional study. The associations of CKD defined by each of the three markers alone and in combination with moderate DR were examined using logistic regression models adjusted for potential confounding factors including duration of diabetes, smoking, body mass index, systolic blood pressure, and HbA1c. *Results.* The prevalence of moderate DR was significantly higher among those with CKD defined by triple markers (41.1%) compared to CKD defined separately by creatinine (26.6%), cystatin C (20.9%), and albuminuria (23.4%). People with CKD defined by triple markers had a fourteenfold higher odds of moderate DR (OR (95% CI) = 13.63 (6.08–30.54)) compared to those without CKD by any marker. Nearly half (48.7%) of participants with cystatin C ≥ 1.12 mg/L have moderate DR. *Conclusions.* CKD defined by a triple marker panel was strongly associated with moderate DR in this Asian population with diabetes.

## 1. Introduction

Diabetic retinopathy (DR), a microvascular complication of diabetes mellitus (DM), is a global public health problem affecting an approximate of 93 million people worldwide, 28 million of whom suffer from vision threatening DR [1]. In Asian countries, the prevalence of DR among those with diabetes ranges from 15.8 to 43.1% [2–4]. With the rapidly rising prevalence of DM in Asian countries like China and India [5] and an ageing population, the socioeconomic burden of DR is likely to increase exponentially in the near future. Chronic kidney disease (CKD), another major complication of diabetes, is also on the rise worldwide. In diabetic patients, diabetic nephropathy (DN) defined by the presence of albuminuria has been shown to be associated with DR in several studies [6–10]. The concordance between DN and DR could be explained by the sharing of common risk factors including glycemic control, duration of diabetes, blood pressure control, and common pathogenic pathways between the two microvascular complications [11]. In addition, genetic risk factors have also been shown to contribute to the concordance between DN and DR [12, 13]. Genetic variants such as* CPVL/CHN, rs 39059,* associated with DN [14, 15] have also been shown to be associated with DR [16, 17]. The cannabinoid type 1 receptor gene polymorphism (G1359A in CNR1 gene), shown to be significantly associated with DR and DN in type 2 diabetics [18], suggests another pathogenic link between nephropathy and retinopathy. However, this concordance between albuminuria and DR has been shown to be stronger in type 1 diabetic patients. In type 2 diabetes, although similar association has been documented [6, 9, 10, 19], the discordance between the two has been shown to be frequent due to the coexistence of nondiabetic kidney disease. Recent studies have shown that, in diabetic patients, CKD is less likely to be associated with DR in the absence of albuminuria [6, 9, 10].

In current clinical practice, CKD is defined by glomerular filtration rate estimated from serum creatinine. Recently, serum cystatin C, an alternative marker of kidney function, either alone or in combination with serum creatinine and/or albuminuria has been shown to be a better predictor of adverse outcomes than serum creatinine alone [20–25]. Serum cystatin C is a cysteine protease inhibitor found in virtually all human tissues and body fluids, which, in contrast to creatinine, is less affected by age, race, or muscle mass [24, 26]. A clinic based study from China has shown that elevated levels of cystatin C are associated with severity of DR and are an independent risk factor for DR along with diabetes duration and HbA1c levels [27]. A double marker approach combining serum creatinine and albuminuria has been shown to be strongly associated with DR compared to creatinine alone in recent studies [6, 9, 10]. However, the association of CKD defined by a triple marker panel including cystatin C in addition to creatinine and albuminuria with DR has not been evaluated before. The association with DR could be stronger either by better classification of patients into CKD categories or by virtue of being markers for different pathologic processes in DR, independent of GFR mechanisms. In this context, we examined the association of CKD defined by triple markers with diabetes-specific moderate retinopathy in a population-based sample of Asian adults with diabetes. The aim of this study was to determine whether CKD defined by all triple markers (creatinine, albuminuria, and cystatin C) was more strongly associated with moderate DR compared to each marker in isolation or in dual combination.

## 2. Materials and Methods

### 2.1. Study Population and Design

**Figure 1:**
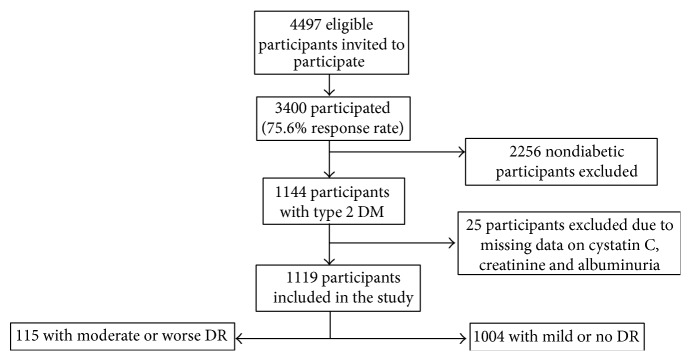
Flow diagram of study enrolment. All participants of Indian ethnicity were eligible unless he/she had moved from the residing address, had not lived at the official address in the past six months, or was terminally ill (e.g., cancer) or deceased.

The current study is based on data derived from the Singapore Indian Eye Study (SINDI), a population-based cross-sectional study of 3,400 Indian adults aged 40–80 years living in Singapore between 2007 and 2009. Details of the study design, sampling plan, and methodology have been reported elsewhere [28]. In brief, 6,350 adults were selected by an age-stratified random sampling method from the computer generated random list of 11,616 Indian names provided by the Ministry of Home Affairs. Of the 4,497 eligible participants, 3,400 participated in the study (75.6% response rate). Of these, 1,144 participants had diabetes mellitus defined as random glucose of 11.1 mmol/L or more, use of diabetic medication, or a physician diagnosis of diabetes mellitus or HbA1c > 6.5% (47.5 mmol/mol). After excluding those with missing information on cystatin C, creatinine, albuminuria, and other variables included in the multivariable model (*n* = 25), 1,119 participants with diabetes mellitus were included in the current study (Figure 1). The study was conducted in accordance with the Declaration of Helsinki and approval for the study was granted by the National Healthcare Group (2012/00291) and Singhealth (2012/377/A) Institutional Review Boards. Written informed consent was obtained from all participants before enrolment.

### 2.2. Study Procedures

Standardized systemic and ocular examinations, interviewer-administered questionnaires, and standard blood investigations were conducted for all participants. A detailed interviewer-administered questionnaire was used to collect relevant demographic data and medical history from all participants. Alcohol drinkers were defined by the consumption of alcohol at least once a week. Blood pressure was measured with a digital automatic blood pressure monitor (Dinamap model Pro Series DP110X-RW, 100V2; GE Medical Systems Information Technologies, Inc., Milwaukee, WI) after the participants were seated for at least 5 minutes. Venous blood samples were collected for biochemistry tests, including serum lipids (total cholesterol, high-density lipoprotein cholesterol, and low-density lipoprotein cholesterol), glycosylated hemoglobin A1c (HbA1c), creatinine, and random glucose. The average of the 2 systolic and diastolic blood pressure measurements was used as the systolic and diastolic blood pressure value. Hypertension was defined as systolic blood pressure of 140 mmHg or more or diastolic blood pressure of 90 mmHg or more or self-reported physician-diagnosed hypertension. Diabetes mellitus was defined as random glucose of 11.1 mmol/L or more, use of diabetic medication, or a physician diagnosis of diabetes mellitus [29, 30]. We did not differentiate between type 1 or type 2 diabetes. However, since 95% of our participants reported having their diabetes diagnosed after the age of 35 years, we assume that the majority had type 2 diabetes.

### 2.3. Assessment of DR

Fundus photography was performed using a digital nonmydriatic retinal camera (Canon CRDGi with a 20Diopter SLR backing, Canon, Japan) using early treatment for diabetic retinopathy study (ETDRS) standard field 1 (centered on the optic disc) and ETDRS standard field 2 (centered on the fovea). DR was evaluated following a standard protocol based on retinal photographs which were graded according to a modified scale from the Airlie House Classification System by trained graders [31]. Moderate DR was defined to be hemorrhages and/or microaneurysms ≥ standard photograph 2A and/or soft exudates, venous beading, or intraretinal microvascular abnormalities definitely present and definition not met for severe nonproliferative retinopathy, early proliferative retinopathy, or high-risk proliferative retinopathy. Severe DR, including both proliferative and nonproliferative severe DR, was defined as soft exudates, venous beading, and intraretinal microvascular abnormalities all definitely present in at least two of fields four through seven or two of the preceding three lesions present in at least two of fields four through seven and hemorrhages and microaneurysms present in these four fields, equaling or exceeding standard photo 2A in at least one of them or intraretinal microvascular abnormalities present in each of fields four through seven equaling or exceeding standard photograph 8A in at least two of them or presence of new vessels [31]. Any DR was defined as ETDRS severity score ≥ 14 in at least one eye and moderate DR was defined as ETDRS severity score ≥ 43 in at least one eye. The definition of DR severity was based on the worse eye. Moderate and severe DR were combined into a single category (moderate) as the number of subjects with severe DR were too few for an adequately powered statistical analysis.

### 2.4. Assessment of Chronic Kidney Disease (CKD)

Based on each individual marker, chronic kidney disease was defined as eGFR of <60 mL/minute/1.73 m^2^, using the US National Kidney Foundation Kidney Disease Outcome Quality Initiative Working Group definition [32]; GFR was estimated from the serum creatinine concentration (eGFR_cr_) [33] and serum cystatin C (eGFR_cys_) [34] using the recently developed CKD epidemiology collaboration (CKD-EPI) equations. We have earlier shown the prevalence of CKD by CKD-EPI equation to be similar to that of MDRD (modification of diet in renal disease) equation in all three major ethnic groups (Chinese, Malays, and Indians) in Singapore [35]. Creatinine concentrations were determined by the Jaffe method on the Beckman DxC800 analyzer with manufacturer provided calibrators traceable to SRM 967 [36]. Presence of albuminuria was defined as a urinary albumin : creatinine ratio of 30 mg/g [9, 37]. Spot untimed urine samples were collected for measurement of albumin and creatinine. Albumin was measured in mg/L and creatinine in mmol/L. The concentration ratio of urinary albumin to creatinine expressed in *μ*g/mg was used to estimate the total daily albumin excretion. Cystatin C was measured using particle-enhanced turbidimetric assay, and urine albumin was measured using a PEG-enhanced immunoturbidimetric method on the Siemens Advia platform at NUHS laboratory.

### 2.5. Statistical Analysis

Statistical analysis was performed with STATA version 13.0 (Texas, USA). A *P* value of <0.05 was considered statistically significant. For univariate analysis, the Chi square test was performed for categorical variables and the independent *t* test for continuous variables. The associations of CKD defined by each of the three markers alone and in combination (eGFR_cr_ < 60 + eGFR_cys_ < 60 + albuminuria) with moderate DR were examined using logistic regression models: first adjusted for age and gender, followed by adjustment for potential confounding factors including duration of diabetes, smoking, body mass index (BMI), systolic blood pressure, alcohol consumption, total cholesterol, and high-density lipoprotein cholesterol. Odds ratio (OR) and 95% confidence interval (CI) were reported for the associations of CKD markers with moderate DR. Logistic regression models consisting of CKD markers in isolation and in combination were checked for goodness of fit with the Hosmer-Lemeshow test. To assess the performance of triple marker model including cystatin C in predicting moderate DR, we constructed receiver operating characteristic (ROC) curves for each marker alone and in combination. The area under curve (AUC) for each model was compared with the null model without any CKD marker. In order to examine whether cystatin C is associated with DR, independent of kidney function, we also examined the association between serum cystatin C levels (continuously and in quartiles) and moderate DR in separate logistic regression models. The models were similar to the main analysis, except for additional adjustment for eGFR_cr_ and albuminuria in the multivariable models. Finally, we repeated the main analysis after excluding participants who reported having their diabetes diagnosed before the age of 35 years.

## 3. Results

A total of 1119 subjects with diabetes were enrolled into the study. Of these, 115 (10.3%) had moderate DR in at least one eye.  Table 1 shows the characteristics of subjects with and without moderate DR. Persons with moderate DR had higher prevalence of hypertension, had higher levels of systolic BP, BMI, blood glucose, HbA1c, and ACR and longer duration of diabetes and had lower levels of eGFR (both eGFR_cr_ and eGFR_cys_). The prevalence of CKD defined by creatinine, cystatin C, and albuminuria in our cohort was 11.4%, 20.1%, and 29.0%, respectively. In subjects with CKD defined by eGFR_cr_ < 60 mL/min/1.73 m^2^, 86.7% (*n* = 111) also had eGFR_cys_ < 60 mL/min/1.73 m^2^ and 46.0% (*n* = 59) had ACR ≥ 30 mg/g. In subjects without CKD defined by eGFR_cr_ (*n* = 991), 11.5% (*n* = 114) had eGFR_cys_ < 60 mL/min/1.73 m^2^ and 34.4% (*n* = 341) had ACR ≥ 30 mg/g. Five percent (*n* = 56) of adults had CKD defined by all 3 markers.

Table 2 shows the results of multivariate analysis performed to assess the association between each marker in isolation and in combination. The prevalence of moderate DR was higher among those with CKD-eGFR_cr_ (26.6%) followed by albuminuria (23.4%) and CKD-eGFR_cys_ (20.9%). In separate models, CKD-eGFR_cr_, CKD-eGFR_cys_, and albuminuria were significantly associated with DR with ORs ranging from 2.50 for albuminuria to 5.21 for CKD-eGFR_cr_. Subjects with CKD defined by triple markers were fourteen times more likely to have moderate DR (OR (95% CI) = 13.63 (6.08–30.54)) compared to those without CKD defined by any marker.

**Figure 2:**
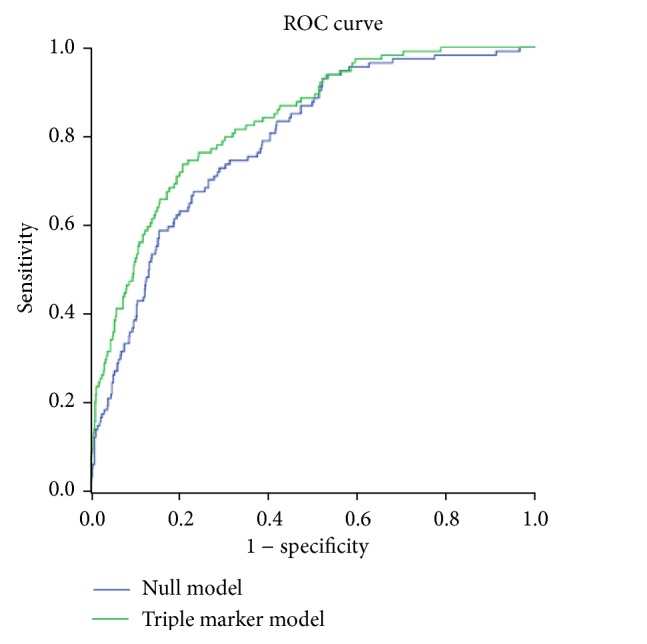
Receiver operating characteristic (ROC) curves comparing the null model (age, gender, body mass index, systolic BP, HbA1c, and diabetes duration) with the triple marker model (eGFR_cr_ + eGFR_cys_ + albuminuria, in addition to the variables included in the null model).

Table 3 shows the AUC for diagnostic models consisting of CKD markers in isolation and in combination, compared to the null model without CKD markers. Diagnostic models containing CKD defined by any of the 3 markers (creatinine, cystatin C, and ACR) alone were not significantly better than the null model (*P* = 0.09, 0.07, and 0.07, resp.). In the models combining 2 markers, CKD defined by a combination of creatinine and albuminuria (AUC 0.828, *P* = 0.01) or a combination of cystatin C and albuminuria (AUC 0.828, *P* = 0.02) had better discrimination than the null model. The model using triple markers had the highest AUC of 0.834 and was strongly significant for better discrimination than the null model (*P* = 0.0089, Figure 2). When the triple marker model was compared to single and double marker models, incremental diagnostic value was significantly better than the cystatin C model (*P* = 0.035) but not for the other models. The association of serum cystatin C levels with moderate DR is shown in Table 4. The prevalence of moderate DR increased with increasing quartiles of cystatin C with nearly half of the participants in the highest quartile having moderate DR (48.7%). Cystatin C was significantly associated with moderate DR in both quartile and continuous analysis independent of eGFR_cr_ and ACR. Finally, repeating the main analysis in Table 2, after excluding those with diabetes diagnosed before the age of 35 years (*n* = 53 excluded), did not alter the effect estimates. For example, compared to those without CKD by any marker, the OR (95% CI) of moderate DR was 2.23 (1.19–4.20) by any one marker; 6.99 (3.14–15.58) by any 2 markers; and 13.04 (5.67–30.00) by any 3 markers (data not shown in the table).

## 4. Discussion

In a population-based sample of Indian adults with diabetes, CKD defined by all 3 markers including eGFR_cr_, eGFR_cys_, and albuminuria was strongly associated with moderate DR independent of other cardiovascular risk factors. In addition, elevated levels of cystatin C (≥1.12 mg/L) were associated with more than 3-fold odds of moderate DR independent of albuminuria and eGFR_cr_.

The prevalence of CKD in the current study including persons with diabetes was 11.4% by eGFR_cr_ and 20.1% by eGFR_cys_. 5% had CKD by the triple marker panel. This is similar to previous reports from the US. In the NHANES cohort, the prevalence of reduced kidney function was 16.5% using eGFR_cr_ and 22% using eGFR_cys_ among persons with diabetes [38]. In the REGARDS cohort consisting of subjects with and without diabetes, 12.9% had reduced kidney function using eGFR_cys_, 10.9% had reduced kidney function using eGFR_cr_, and 14.8% had albuminuria. Only 3.3% had CKD defined by all 3 markers [23].

Several studies have shown associations between CKD defined by a single marker including creatinine [10, 39–41] or albuminuria [10, 41] and DR. Few previous studies have shown that CKD defined by double markers including eGFR_cr_ and albuminuria is strongly associated with DR compared to eGFR_cr_ alone. Chen et al. found CKD defined by eGFR_cr_ to be associated with DR only in the presence of albuminuria [6]. Similarly, in a recent cross-sectional study involving a multiethnic Asian population, we found that CKD was associated with DR only in the presence of albuminuria [9]. Penno et al. found albuminuric CKD phenotypes with reduced eGFR_cr_ to be strongly associated with advanced DR (OR 2.97), compared to both albuminuric CKD without reduced eGFR_cr_ and nonalbuminuric CKD phenotypes [10].

Recently, CKD outcome studies have established that cystatin C and albuminuria can improve risk stratification among persons with CKD defined by eGFR_cr_, with respect to mortality risk, cardiovascular disease, heart failure, and end stage renal disease [22, 42–44]. In the reasons for geographic and racial differences in stroke (REGARDS) cohort, all-cause mortality was higher in persons with CKD defined by eGFR_cr_ who also had albuminuria [43] or CKD based on eGFR_cys_ [22]. More recently, Peralta et al. evaluated a triple marker approach for the detection and classification of CKD using creatinine, cystatin C, and ACR [23]. They found the highest risk for all-cause mortality and incident end-stage renal disease among those with CKD defined by all markers compared to those with CKD defined by creatinine alone. Their findings confirmed that a triple marker approach for risk classification in persons with CKD increased the predictive accuracy for all-cause mortality and end-stage renal disease compared to the traditional creatinine based definition of CKD alone. In the current study, for the first time, we have shown a triple marker panel of defining CKD to have a stronger association with DR compared to single or double marker panels. Whether the triple marker approach also increases the predictive value for incident DR or progression of DR remains to be determined in future longitudinal studies.

Compared to CKD defined by serum creatinine, CKD defined by serum cystatin C appears to better predict and risk stratify patients with diabetes for end stage renal disease [20, 21]. In the National Health and Nutrition Examination Survey (NHANES), Tsai et al. found a higher prevalence of reduced kidney function among persons with diabetes using eGFR_cys_ compared to eGFR_cr_ [38]. In the same study, eGFR_cys_ was strongly associated with diabetic complications, including DR [38]. It is possible that cystatin C could also be a better predictor for DR compared to eGFR_cr_ and albuminuria, related to shared pathogenic pathways between retinopathy and cystatin C [45]. In He et al.'s study, the prevalence of sight threatening DR (STDR) was highest in the 4th quartile (~60%, *P* < 0.01) and cystatin C was found to be an independent risk factor for STDR together with HbA1c and diabetes duration. An 11-fold risk of STDR was conferred when serum cystatin C levels were more than 1.25 mg/L [27]. In our study, higher levels of serum cystatin C showed a significant association with DR independent of eGFR_cr_ and albuminuria. This suggests that cystatin C may also play a direct role in DR, independent of kidney function, by promoting vascular endothelial growth factor (VEGF) driven angiogenesis [45]. Further studies are required to determine the concordance of cystatin C defined CKD phenotypes with DR.

The relatively small number of subjects with CKD defined by triple markers is a limitation of our study, resulting in an imprecise confidence interval estimate (OR (95% CI) = 13.63 (6.08–30.54)). However, despite the smaller sample size, our effect estimates for CKD by triple marker remained highly statistically significant due to a large effect size. Increasing the sample size can only strengthen our conclusions. The cross-sectional nature of our study is another limitation of our study. Although a prospective study would be ideal to assess the risk of DR progression, the use of triple markers to predict the presence of moderate to severe DR may still prove to be useful to patients and clinicians without easy access to fundus photography or an ophthalmologist.

In conclusion, CKD defined by a triple marker panel was strongly associated with moderate DR in Indian adults with diabetes. If confirmed by future prospective studies and in other populations, a triple marker approach may have implications on changing clinical practice to incorporate cystatin C to improve risk stratification of DR in persons with CKD. This group of high-risk individuals may benefit from closer surveillance and more timely intervention before the onset of irreversible sight threatening complications.

## Figures and Tables

**Table 1:** Characteristics of participants by moderate DR status.

	No or mild DR	Moderate DR	*P* value^*^
	(*n* = 1,004)	(*n* = 115)	
Age (years)	59.6 (9.8)	60.6 (8.2)	0.29
Women, %	454 (45.2)	49 (42.6)	0.59
Current smokers, %	131 (13.05)	9 (7.83)	0.11
Alcohol drinkers, %	140 (13.9)	10 (8.7)	0.12
Hypertension, %	686 (68.5)	92 (80)	0.01
Systolic blood pressure (mm Hg)	139 (19)	145 (21)	0.001
Diastolic blood pressure (mm Hg)	78 (10)	76 (10)	0.08
BMI (kg/m^2^)	27.14 (5.03)	25.53 (4.39)	0.001
LDL-cholesterol	3.07 (0.95)	3.02 (0.10)	0.63
HDL-cholesterol	1.02 (0.29)	1.07 (0.41)	0.17
Blood glucose, mmol/L	9.51 (4.22)	12.08 (6.29)	<0.001
HbA1c, mmol/mol	59	69	<0.001
HbA1c, (%)	7.5 (1.5)	8.5 (2.0)	<0.001
Duration of diabetes, years	9.5 (8.2)	17.0 (10.5)	<0.001
eGFR_cr_ (mL/min/1.73 m^2^)	87 (18)	73 (26)	<0.001
eGFR_cys_ (mL/min/1.73 m^2^)	82 (22)	68 (27)	<0.001
ACR (mg/g)	57.5 (213)	638.7 (3055)	<0.001

DR: diabetic retinopathy; BMI: body mass index; LDL: low density lipoprotein; HDL: high density lipoprotein; eGFR_cr_: estimated glomerular filtration rate by serum creatinine; eGFR_cys_: estimated glomerular filtration rate by serum cystatin C; ACR: albumin to creatinine ratio.

Data presented are proportions or means and standard deviation as appropriate for the variable.

^*∗*^ *P* value represents difference in characteristics by moderate DR status by analysis of variance or the chi-square test.

**Table 2:** Association between markers of CKD and moderate DR in the study population.

	*n* (cases)	Prevalence of moderate DR, %	Age, sex adjusted	Multivariable
	1119 (115)		OR (95% CI)	OR (95% CI)^*^
eGFR_cr _				
≥60	991 (81)	8.2	1 (Reference)	1 (Reference)
<60	128 (34)	26.6	4.33 (2.66–7.04)	5.21 (2.94–9.21)
eGFR_cys _				
≥60	894 (68)	7.6	1 (Reference)	1 (Reference)
<60	225 (47)	20.9	3.75 (2.37–5.93)	5.27 (3.08–9.02)
Albuminuria				
No	680 (39)	5.7	1 (Reference)	1 (Reference)
Yes	324 (76)	23.4	4.05 (2.69–6.10)	2.50 (1.58–3.96)
CKD				
None by all	581 (24)	4.1	1 (Reference)	1 (Reference)
Any 1 marker	379 (48)	12.7	3.59 (2.15–6.01)	2.33 (1.33–4.11)
Any 2 markers	103 (20)	19.4	7.31 (3.66–14.60)	8.00 (3.72–17.17)
All 3 markers	56 (23)	41.1	19.66 (9.68–39.91)	13.63 (6.08–30.54)

Abbreviations: CI, confidence interval; OR, odds ratio; SD, standard deviation.

^*^Model adjusted for age (years), gender (men, women), body mass index (kg/m^2^), systolic blood pressure (mm Hg), HbA1c (%) and diabetes duration (years).

**Table 3:** Area under curve (AUC) for markers of CKD in isolation and in combination for discriminating persons with/without moderate DR.

	AUC	95% CI	*P* ^*∗*^
Null model	0.790	0.748–0.832	—
eGFR_cr_ only	0.813	0.775–0.852	0.09
eGFR_cys_ only	0.816	0.776–0.857	0.07
Albuminuria only	0.808	0.768–0.849	0.07
eGFR_cr_ + eGFR_cys_	0.821	0.781–0.860	0.05
eGFR_cr_ + albuminuria	0.828	0.791–0.864	0.01
eGFR_cys_ + albuminuria	0.828	0.789–0.868	0.015
eGFR_cr_ + eGFR_cys_ + albuminuria	0.834	0.795–0.871	0.0089

CI: confidence interval.

Null model: age, gender, body mass index, systolic BP, HbA1c, and diabetes duration.

^*∗*^ *P* value, compared with null model.

**Table 4:** Association between serum cystatin C and moderate DR.

Cystatin C	Prevalence of DR	Age-gender adjusted OR	^*^Multivariable adjusted OR
		(95% CI)	(95% CI)
Q1 (<0.82)	19 (16.5%)	1 (Reference)	1 (Reference)
Q2 (0.82–0.94)	21 (18.3%)	1.08 (0.57–2.07)	1.05 (0.51–2.16)
Q3 (0.95–1.11)	19 (16.5%)	0.99 (0.50–1.94)	1.00 (0.46–2.17)
Q4 (1.12–5.07)	56 (48.7%)	3.51 (1.94–6.38)	3.38 (1.55–7.38)
Per SD increase	—	1.92 (1.59–2.33)	1.90 (1.37–2.61)

DR: diabetic retinopathy; Q: quartiles; SD: standard deviation.

^*^Model adjusted for age (years), gender (men, women), body mass index (kg/m^2^), systolic blood pressure (mm Hg), HbA1c (%), diabetes duration (years), albumin creatinine ratio (mg/g), and eGFR_cr_ (mL/min/1.73 m^2^).
